# A distribution-free and analytic method for power and sample size calculation in single-cell differential expression

**DOI:** 10.1093/bioinformatics/btae540

**Published:** 2024-09-04

**Authors:** Chih-Yuan Hsu, Qi Liu, Yu Shyr

**Affiliations:** Department of Biostatistics, Vanderbilt University Medical Center, Nashville, TN 37203, United States; Center for Quantitative Sciences, Vanderbilt University Medical Center, Nashville, TN 37203, United States; Department of Biostatistics, Vanderbilt University Medical Center, Nashville, TN 37203, United States; Center for Quantitative Sciences, Vanderbilt University Medical Center, Nashville, TN 37203, United States; Department of Biostatistics, Vanderbilt University Medical Center, Nashville, TN 37203, United States; Center for Quantitative Sciences, Vanderbilt University Medical Center, Nashville, TN 37203, United States

## Abstract

**Motivation:**

Differential expression analysis in single-cell transcriptomics unveils cell type-specific responses to various treatments or biological conditions. To ensure the robustness and reliability of the analysis, it is essential to have a solid experimental design with ample statistical power and sample size. However, existing methods for power and sample size calculation often assume a specific distribution for single-cell transcriptomics data, potentially deviating from the true data distribution. Moreover, they commonly overlook cell–cell correlations within individual samples, posing challenges in accurately representing biological phenomena. Additionally, due to the complexity of deriving an analytic formula, most methods employ time-consuming simulation-based strategies.

**Results:**

We propose an analytic-based method named scPS for calculating power and sample sizes based on generalized estimating equations. scPS stands out by making no assumptions about the data distribution and considering cell–cell correlations within individual samples. scPS is a rapid and powerful approach for designing experiments in single-cell differential expression analysis.

**Availability and implementation:**

scPS is freely available at https://github.com/cyhsuTN/scPS and Zenodo https://zenodo.org/records/13375996.

## 1 Introduction

Single-cell transcriptome has emerged as a powerful tool for characterizing cellular heterogeneity within complex tissues and modelling cell trajectories during developmental process (Fu *et al.* 2023, Wang *et al.* 2023, Kirschenbaum *et al.* 2024). Identifying differentially expressed genes (DEGs) in single-cell transcriptome is crucial for unveiling cell type-specific responses to various treatments or biological conditions. To ensure the robustness and reliability of the analysis, it is essential to have a robust experimental design with an adequate sample size to reach a certain statistical power. Current methods for power analysis and sample size calculation address two distinct research questions: one focusing on the comparison of cell subpopulations and the other on the detection of DEGs (Jeon *et al.* 2023).

Several methods have been developed for power and sample size calculation specifically focusing on DEG analysis (Table 1). Many of these methods adopt simulation-based strategies, including powsimR (Vieth *et al.* 2017), POWSC (Su *et al.* 2020), scDesign (Li and Li 2019), and Hierarchicell (Zimmerman and Langefeld 2021). powsimR and Hierarchicell assume gene expressions follow negative binomial (NB) distributions, while POWSC assumes data follow a mixture of zero-inflated Poisson and log-normal Poisson distributions, and scDesign assumes log-transformed normalized data follow a mixture of gamma and normal distributions. Notably, only Hierarchicell accounts for expression correlation among cells within individual samples. However, these simulation-based approaches are computationally intensive and time-consuming. A recent method, scPower (Schmid *et al.* 2021), introduces an analytic formula for power calculation based on Zhu and Lakkis’s DEG analysis method (Zhu and Lakkis 2014). scPower employs the pseudobulk approach, assuming pseudobulk counts follow NB distributions. Being an analytic-based approach, scPower is significantly faster than simulation-based methods. It is important to note that scPower overlooks cell correlations within samples by assuming cell independence. This contradicts biological reality, where cells from the same sample share a common background and are not statistically independent; therefore, gene expression from cells within the same sample should be more correlated with each other than with cells from different samples (Zimmerman *et al.* 2021). In all five single-cell transcriptomics datasets we investigated, e.g. we observed intra-sample correlations (ICCs) in all cell types from various tissues (Supplemental Fig. S1 and Table S1).

**Table 1.** Comparison of power and sample size calculation methods for single-cell DEG analysis.

	Data distribution	Cell–cell correlations	Analytic-based
**powsimR**	Negative binomial	No	No
**POWSC**	A mixture of zero-inflated Poisson and log-normal Poisson distributions	No	No
**scDesign**	A mixture of gamma and normal distributions on log-transform normalized data	No	No
**Hierarchicell**	Negative binomial	Yes	No
**scPower**	Negative binomial	No	Yes
**scPS**	Distribution-free	Yes	Yes

**Figure 1.**
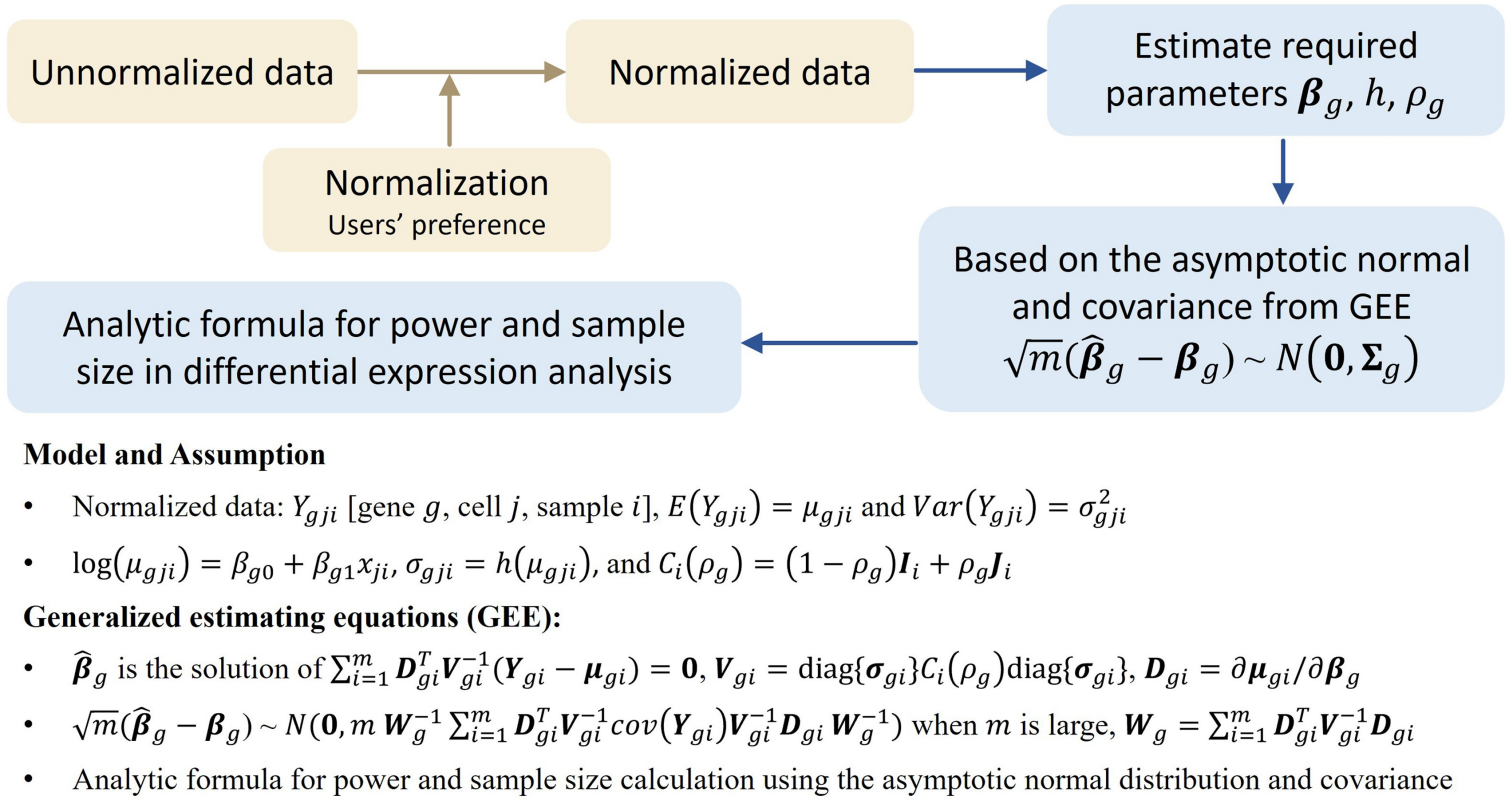
The schema of scPS. scPS starts with the normalized data collected from a pilot study, estimates the required parameters, and calculates power and determines required sample sizes based on the distribution-free GEE approach

The assumptions of current methods regarding data distributions and their neglect of cell–cell correlations pose limitations on their applicability and accuracy. To address these challenges, we propose a novel analytic method, scPS, designed for calculating power and sample size in the detection of DEGs in single-cell transcriptomics analysis. scPS utilizes the distribution-free generalized estimating equations (GEEs) approach (Liang and Zeger 1986) (Fig. 1). This method begins with normalized pilot data, allowing flexibility in normalization methods and making no assumptions about data distributions. In contrast to other methods that assume a specific data distribution and learn the parameters of the distribution from pilot data (Table 1), scPS is distribution-free and only learns the mean-variance relationship from pilot data. A given data distribution defines a specific mean-variance relationship, but a given mean-variance relationship does not define a specific distribution. Thus, scPS is less restrictive than existing methods in terms of data distribution assumptions. Importantly, scPS accounts for cell–cell correlations within individual samples (Table 1) rather than assuming cell independence. If there is no ICC, scPS simplifies to a cell–cell independence model. As an analytic method, scPS is computationally fast for calculating power and sample size, including the number of samples per group and the number of cells per sample. Additionally, scPS is applicable to both independent two-group and paired-group experimental designs.

## 2 Methods

### 2.1 Model and assumption

Given one cell type of interest, $Y_{\mathrm{gji}}$ is the normalized expression level of gene g in cell j from sample i, g=1,…,G (genes), $j=1,\ldots ,n_{i}$ (cells from the cell type of interest), and i=1,…,m (samples, denoting subjects/patients). The mean and the variance of $Y_{\mathrm{gji}}$ is denoted by $\mu_{\mathrm{gji}}$ and $\sigma_{\mathrm{gji}}^{2}$, respectively. Let $\log\left(\mu_{\mathrm{gji}}\right)=\beta_{g0}+\beta_{g1}x_{i}$, where $x_{i}$ is a variable to indicate that sample i belongs to the control group or the experimental group, denoted by 0 or 1. $\beta_{g0}$ is the log transformation of the mean expression of gene g in the control group, and $\beta_{g1}$ is the log fold change (logFC) in expression of gene g between the experimental group and the control. We assume the relationship between $\sigma_{\mathrm{gji}}$ and $\mu_{\mathrm{gji}}$ by $\sigma_{\mathrm{gji}}=h(\mu_{\mathrm{gji}})$, where h is a positive function. For example, if $h(\mu_{\mathrm{gji}})=\sqrt{\mu_{\mathrm{gji}}}$, $Y_{\mathrm{gji}}\;$might follow a Poisson distribution, or if $h(\mu_{\mathrm{gji}})=\sqrt{\mu_{\mathrm{gji}}(1+\phi \mu_{\mathrm{gji}})}$ with a constant ϕ>0, $Y_{\mathrm{gji}}\;$might follow a NB distribution. In the calculation of power and sample size, h is estimated from the pilot data. Moreover, we assume $C_{i}\left(\rho_{g}\right)=\left(1-\rho_{g}\right)I_{i}+\rho_{g}J_{i}$ as the correlation matrix of $Y_{gi}={\left(Y_{\mathrm{gji}},j=1,\ldots ,n_{i}\right)}^{T}$, where $I_{i}$ is an identity matrix of dimension $n_{i}$ and $J_{i}$ is a square matrix that all elements are 1 and its dimension is the same as $I_{i}$. $\rho_{g}\;$is the ICC that denotes the cell–cell correlation within a sample for gene g (see details in the Section 2.6).

### 2.2 Generalized estimating equations

Given $Y_{gi}$, $x_{i}$, and the assumed h and $C_{i}$, the estimate ${\hat{\beta }}_{g}={\left({\hat{\beta }}_{g0},\;{\hat{\beta }}_{g1}\right)}^{T}$for the parameter $\beta_{g}={\left(\beta_{g0},\;\beta_{g1}\right)}^{T}$ can be obtained by solving the equation

(1) $$\sum_{i=1}^{m}D_{gi}^{T}V_{gi}^{-1}(Y_{gi}-\mu_{gi})=0,$$

where $D_{gi}=(\partial \mu_{gi}/\partial \beta_{g0},\partial \mu_{gi}/\partial \beta_{g1})$, $V_{gi}=\mathrm{diag}\{\sigma_{gi}\}C_{i}\left(\rho_{g}\right)\mathrm{diag}\{\sigma_{gi}\}$, $\mu_{gi}={\left(\mu_{\mathrm{gji}},j=1,\ldots ,n_{i}\right)}^{T}$, and $\sigma_{gi}={\left(\sigma_{\mathrm{gji}},j=1,\ldots ,n_{i}\right)}^{T}$. Liang and Zeger (1986) showed that under mild regularity conditions,

(2) $$\begin{array}{c} \sqrt{m}({\hat{\beta }}_{g}-\beta_{g})\;\text{approximates}\\ N\left(0,m\;W_{g}^{-1}\sum_{i=1}^{m}D_{gi}^{T}V_{gi}^{-1}c\mathrm{ov}\left(Y_{gi}\right)V_{gi}^{-1}D_{gi}W_{g}^{-1}\right), \end{array}$$

when m is large, where $W_{g}=\;\sum_{i=1}^{m}D_{gi}^{T}V_{gi}^{-1}D_{gi}$. Therefore, we will use the asymptotic normal distribution and the covariance matrix of ${\hat{\beta }}_{g}$ to calculate powers and sample sizes (m and $n_{i}$). To simplify, we assume an equal number of cells for m samples, i.e. $n_{i}=n$ for all i. In practice, scPS includes a cell-ratio parameter ($r_{c}$) to regulate the ratio of cells between the experimental and control groups, allowing for a different number of cells in each group. Generally, an experimental design with balanced cell abundances between two groups ($r_{c}=1$) achieves the highest power (detailed guidance in the scPS GitHub).

### 2.3 Power for single gene comparison

With the inputs of $\beta_{g}$, $\rho_{g}$, and h, the power to detect the DEG g between two groups is given by

(3) $$\begin{array}{c} \xi_{g}\left(m,n,\beta_{g},\rho_{g},h,\alpha \right)\\ =1+\Phi \left(-z_{1-\alpha /2}-\frac{\beta_{g1}}{\sqrt{V\mathrm{ar}\left({\hat{\beta }}_{g1}\right)}}\right)-\Phi \left(z_{1-\alpha /2}-\frac{\beta_{g1}}{\sqrt{V\mathrm{ar}\left({\hat{\beta }}_{g1}\right)}}\right), \end{array}$$

when m, n, and a significance level of α are given. Φ is the cumulative distribution function (CDF) of the standard normal distribution, $z_{1-\alpha /2}=\Phi^{-1}(1-\alpha /2)$, and $\mathrm{cov}\left(Y_{gi}\right)$ in the asymptotic covariance matrix is substituted by $V_{gi}$. In those single-cell transcriptomic experiments with small sample sizes, it is necessary to revise the power calculation originally based on large sample properties. To adjust for small sample sizes, we adopt the approach mentioned in Mancl and DeRouen (2001), which uses a Student’s *t*- or F-distribution instead of the asymptotic normal (or chi-square) distribution and considers the number of subjects minus the number of coefficients as the degrees of freedom. Accordingly, we replace Φ with $t_{m-2}$, where $t_{m-2}$ is the CDF of the Student’s *t*-distribution with m−2 degrees of freedom. Moreover, various combinations of m and n may yield the same power. The optimal m and n can be further decided by sequencing cost. scPS provides a function to find an optimal combination of m and n that maximizes power under a predetermined budget with a given cost function or minimizes cost while achieving a pre-determined power at a given cost function. For example, given $C\left(m,\;n\right)=mn$, the optimal $m^{*}$ and $n^{*}$ maximize power while ensuring $C\left(m^{*},\;n^{*}\right)+C\left(m^{*}\times r,\;n^{*}\times r_{c}\right)$ remains within the predetermined budget, where r and $r_{c}$ denote the ratio of sample sizes and the ratio of cell numbers, respectively, in the experimental group compared to the control group. Generally, within a predetermined budget, larger sample sizes with fewer cells per sample tend to be more powerful than smaller sample sizes with more cells per sample (detailed guidance in the scPS GitHub).

### 2.4 Power for multiple genes comparison in single cell type

Next, we extend the single-gene comparison to a multiple-gene comparison, mirroring real-world scenarios. We assume K candidate genes in the cell type of interest to be tested, in which there are $K_{0}$ null genes and $K_{1}$ DEGs. While controlling FDR (false discovery rate) at the level of f, the overall power to detect the $K_{1}$ DEGs is given by

(4) $$K_{1}^{-1}\sum_{g\in \mathrm{DEG}}\xi_{g}\left(m,n,\beta_{g},\rho_{g},{h,\alpha }^{*}\right),$$

where $\alpha^{*}\;$satisfies

(5) $$\frac{K_{0}\alpha^{*}}{K_{0}\alpha^{*}+\sum_{g\in \mathrm{DEG}}\xi_{g}\left(m,n,\beta_{g},\rho_{g},h,\alpha^{*}\right)}=f.$$

We use Jung’s formula (Formula (4) of Jung 2005) for FDR calculation without specifying *P*-value distributions. Accordingly, m and n are calculated to achieve the desired overall power. As we mentioned earlier, various combinations of m and n may achieve the same power.

### 2.5 Power for multiple genes comparison in multiple cell types

The power and sample size calculation can be further generalized from one cell type of interest to multiple cell types. Suppose that C cell types, C≥2, are performed in identifying DEGs, where cell proportions of the C cell types are $p_{c},\;c=1,\ldots ,\;C$, respectively. For cell type c, there are $K_{c}$ candidate genes to be tested: $K_{c0}$ null genes and $K_{c1}$ differentially expressed genes ($\mathrm{DE}G_{c}$). With FDR = f, the overall power for the $\sum_{c=1}^{C}K_{c1}$ DEG is given by

(6) $${\left(\sum_{c=1}^{C}K_{c1}\right)}^{-1}\sum_{c=1}^{C}\sum_{g\in \mathrm{DE}G_{c}}\xi_{g}\left(m,n_{c},\beta_{cg},\rho_{cg},{h_{c},\alpha }^{**}\right),$$

where $n_{c}=np_{c}$, and $\beta_{cg}$, $\rho_{cg}$, $h_{c}$ are defined as $\beta_{g}$, $\rho_{g}$, h, respectively, and $\alpha^{**}\;$satisfies

(7) $$\frac{\sum_{c=1}^{C}K_{c0}\alpha^{**}}{\sum_{c=1}^{C}K_{c0}\alpha^{**}+\sum_{c=1}^{C}\sum_{g\in \mathrm{DE}G_{c}}\xi_{g}\left(m,n_{c},\beta_{cg},\rho_{cg},{h_{c},\alpha }^{**}\right)}=f.$$

Note n here is the total number of cells for each sample.

### 2.6 Estimation for the required parameters from pilot data

$\beta_{g}$ , $\rho_{g}$, and h are required parameters of scPS. For each cell type, we estimate $\beta_{g0}$ by the log transformation of the sample mean expression of gene g in the control group, and $\beta_{g1}$ by the log ratio of the sample mean expression in the experimental group to the sample mean expression in the control group or by an experience-based determination for gene g. The function $h\left(\mu_{\mathrm{gji}}\right)=\exp\left(\eta \left(\log\mu_{\mathrm{gji}}\right)\right)$, derived from $\log\left(\sigma_{\mathrm{gji}}\right)=\eta (\log\;\mu_{\mathrm{gji}})$, where the function η is estimated by fitting a natural cubic spline of the log sample SDs on the log sample means among all candidate genes. The 33th and 67th percentiles of the log sample means are specified as the default knots in the spline. $\rho_{g}$, the ICC for gene g, is estimated by averaging the off-diagonal elements of the sample correlation matrix (see Example 3 of Liang and Zeger 1986):

(8) $${\tilde{\rho }}_{g}=\frac{\sum_{i=1}^{m}\left[\sum_{j<j^{\prime }}e_{\mathrm{gji}}e_{gj^{\prime }i}\right]}{\left\{\sum_{i=1}^{m}n_{i}\left(n_{i}-1\right)/2-2\right\}}=\frac{\sum_{i=1}^{m}\left[{\left(\sum_{j=1}^{n_{i}}e_{\mathrm{gji}}\right)}^{2}-\sum_{j=1}^{n_{i}}e_{\mathrm{gji}}^{2}\right]}{\left\{\sum_{i=1}^{m}n_{i}\left(n_{i}-1\right)-4\right\}},$$

where $e_{\mathrm{gji}}=(Y_{\mathrm{gji}}-{\tilde{\mu }}_{gi})/{\tilde{\sigma }}_{gi}$, where ${\tilde{\mu }}_{gi}$ is the sample mean of gene g in group $x_{i}$ and ${\tilde{\sigma }}_{gi}$ is the sample SD of gene g in group $x_{i}$. A $\rho_{g}$ of 0 means that there is no expression correlation of gene g within samples, where a $\rho_{g}$ of 1 indicates gene expression within a sample is identical or perfectly predicted by each other. For multiple cell types, the cell proportion $p_{c}$ is also estimated from pilot data.

### 2.7 Hypothesis testing

We use Wald-type statistic of $\beta_{g1}$ to detect DEGs. We formulate the hypotheses $H_{0}:\beta_{g1}=0$ versus $H_{1}:\beta_{g1}\neq 0$ to determine whether gene g in some cell type of interest is differentially expressed between two groups. Based on the asymptotic distribution of ${\hat{\beta }}_{g}$, under the null hypothesis $H_{0}$, $S={\hat{\beta }}_{g1}/\sqrt{\hat{\mathrm{Var}\left({\hat{\beta }}_{g1}\right)}}$ approximates the standard normal distribution when m is large, where

(9) $$\begin{array}{c} \hat{\mathrm{Var}\left({\hat{\beta }}_{g}\right)}=\;{\hat{W}}_{g}^{-1}\sum_{i=1}^{m}{\hat{D}}_{gi}^{T}{\hat{V}}_{gi}^{-1}\hat{\mathrm{cov}\left(Y_{gi}\right)}{\hat{V}}_{gi}^{-1}{\hat{D}}_{gi}{\hat{W}}_{g}^{-1}\;\mathrm{and}\\ \hat{\mathrm{cov}\left(Y_{gi}\right)}=\left(Y_{gi}-{\hat{\mu }}_{gi}\right){\left(Y_{gi}-{\hat{\mu }}_{gi}\right)}^{T}. \end{array}$$

Thus, $H_{0}$ will be rejected if the *P*-value $=2\left(1-\Phi \left(\left|S\right|\right)\right)<\alpha$, where α is a pre-specified significance level. However, in single-cell transcriptomic experiments, the number of samples m is typically not large although the number of cells per sample is large, leading to an underestimated variance and thus a small *P*-value. Therefore, we replace Φ by $t_{m-2}$, i.e. the modified *P*-value was given by $2\left(1-t_{m-2}\left(\left|S\right|\right)\right)$, as we have done a modification in power calculation. Also, we can combine the bias-corrected covariance estimator (Mancl and DeRouen 2001) for $\mathrm{cov}\left(Y_{gi}\right)$ to further reduce type I error and FDR in multiple gene comparisons. Nevertheless, it is a little conservative when we combine the bias-corrected covariance in multiple gene comparison, according to our simulations (Supplemental Table S2). The Benjamini–Hochberg procedure is applied in multiple gene comparisons to control FDR (Benjamini and Hochberg 1995).

In addition, the numbers of cells within a sample may be quite large and vary with cell types. Directly calculating the inverse of the correlation matrix is computationally intensive. We apply the Sherman–Morrison formula to derive a closed-form expression for the inverse matrix with the special correlation structure we assume here. Furthermore, to speed up the computation for multiple gene comparison, one-step GEE estimator is applied to the estimation of $\beta_{g}$ and $\rho_{g}$ (Lipsitz *et al.* 2017).

### 2.8 Generalized to paired-group comparison

The proposed method can be generalized to paired-group comparisons. We replace $x_{i}$ in $\log\left(\mu_{\mathrm{gji}}\right)=\beta_{g0}+\beta_{g1}x_{i}$ by $x_{ji}$, where $x_{ji}$ is an indicator variable for cell j from sample i. $x_{ji}=0$ and $x_{ji}=1$ denote the two paired groups, respectively. $\beta_{g0}$ is the log transformation of the mean expression of gene g in the control group, and $\beta_{g1}$ is the logFC of expression between two groups for gene g. Similarly, we assume $\sigma_{\mathrm{gji}}=h(\mu_{\mathrm{gji}})$ and $C_{i}\left(\rho_{g}\right)=\left(1-\rho_{g}\right)I_{i}+\rho_{g}J_{i}$, where h is estimated from the pilot data, and the dimensions of $I_{i}$ and $J_{i}$ are the sum of the numbers of cells of interest in two groups. Then, the procedures for power and sample size calculation and the hypothesis testing in the paired-group comparison are similar to those in two independent group comparison.

## 3 Results

### 3.1 scPS estimation is supported by simulations and provides the flexibility of choosing normalization methods

We demonstrated the accuracy of scPS estimation through benchmarking against simulations. In our simulations, we generated single-cell raw counts by following a NB distribution, with mean and variance determined by $\sigma^{2}=\mu (1+\mu /2.3)$. Each simulated cell comprised 2000 genes, exhibiting a cell–cell correlation (ICC) of 0.04. The details for generating single-cell transcriptomics data with ICC were described in Supplemental Materials. Among these genes, 1% were DEGs with |logFC| of 0.693 (FC = 2). The median of the mean expression values of genes was 0.288, with an interquartile range spanning from 0.138 to 0.653. Those parameters were derived from proliferating T cells in patients without COVID-19 infection (Grant *et al.* 2021). We employed four normalization methods, normalized by the total count (RC, relative counts), scran (Lun *et al.* 2016), sctransform (Hafemeister and Satija 2019), and scKWARN (Hsu *et al.* 2024). Each simulation was repeated 100 times.

**Figure 2.**
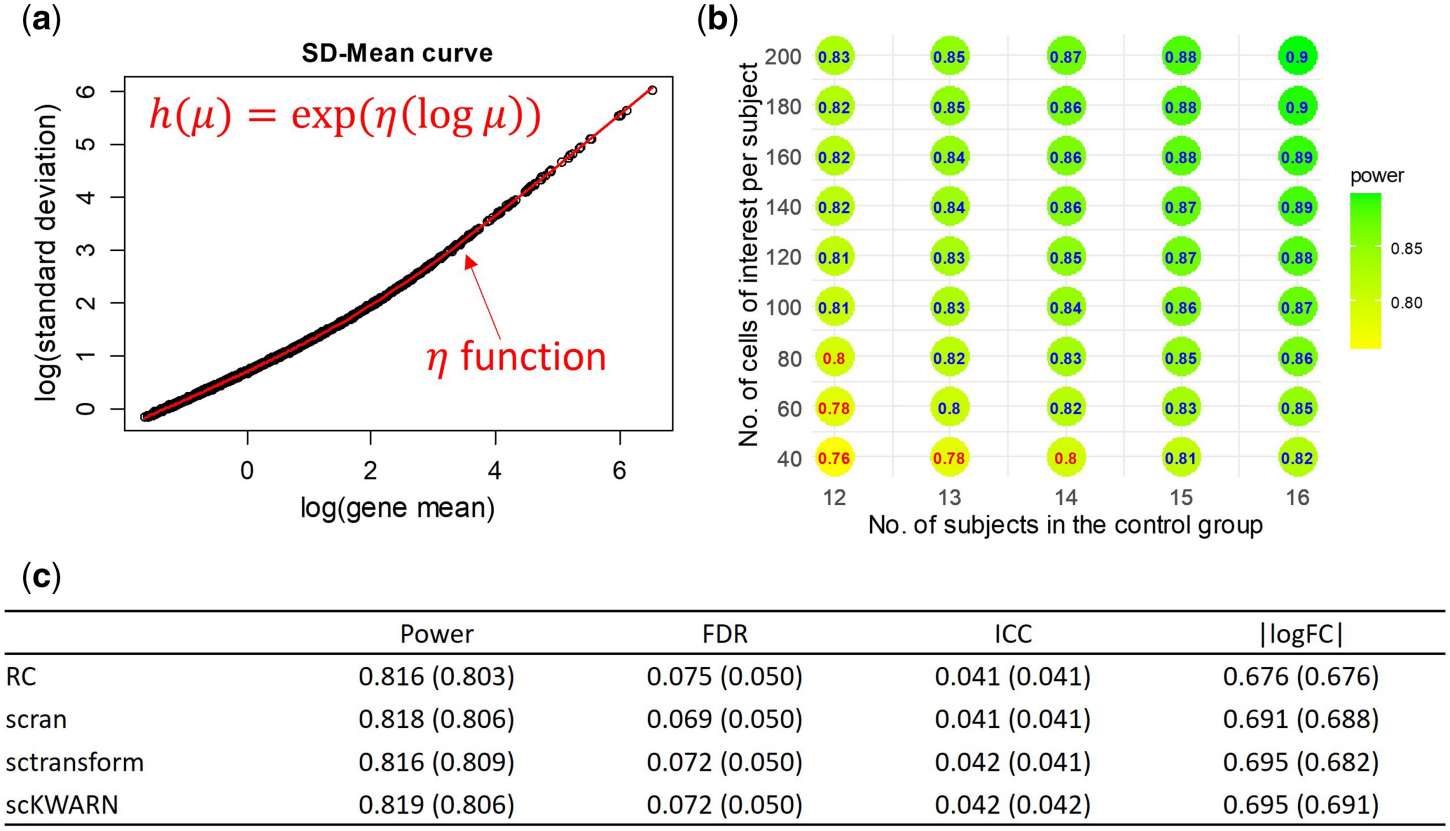
scPS estimations in an independent two-group comparison. (a) Estimation for h function by fitting a composite function on means and SDs of the normalized data. (b) Required sample sizes and cells per sample to achieve a power of 0.80 under FDR = 0.05, based on the parameters estimated from the normalized data. (c) Average empirical overall power, FDR, ICC, |logFC| in 100 simulations, given 13 samples per group and 60 cells per sample. The values in the parentheses are the estimations by scPS

With 13 samples per group and 60 cells per sample, the 100 simulations demonstrated a power of 0.816 and a FDR of 0.075 when RC was employed. In comparison, scPS, starting from the RC-normalized data, obtained a power of 0.803 under the FDR of 0.05, closely mirroring the simulation results (Fig. 2b and c). The required parameters for scPS were estimated from the normalized data, including ICC = 0.04, |logFC| = 0.676, and h function depicted in Fig. 2a (see Section 2.6). Notably, scPS estimation also maintained alignment with simulation results when other three normalization methods were utilized (Fig. 2c).

**Figure 3.**
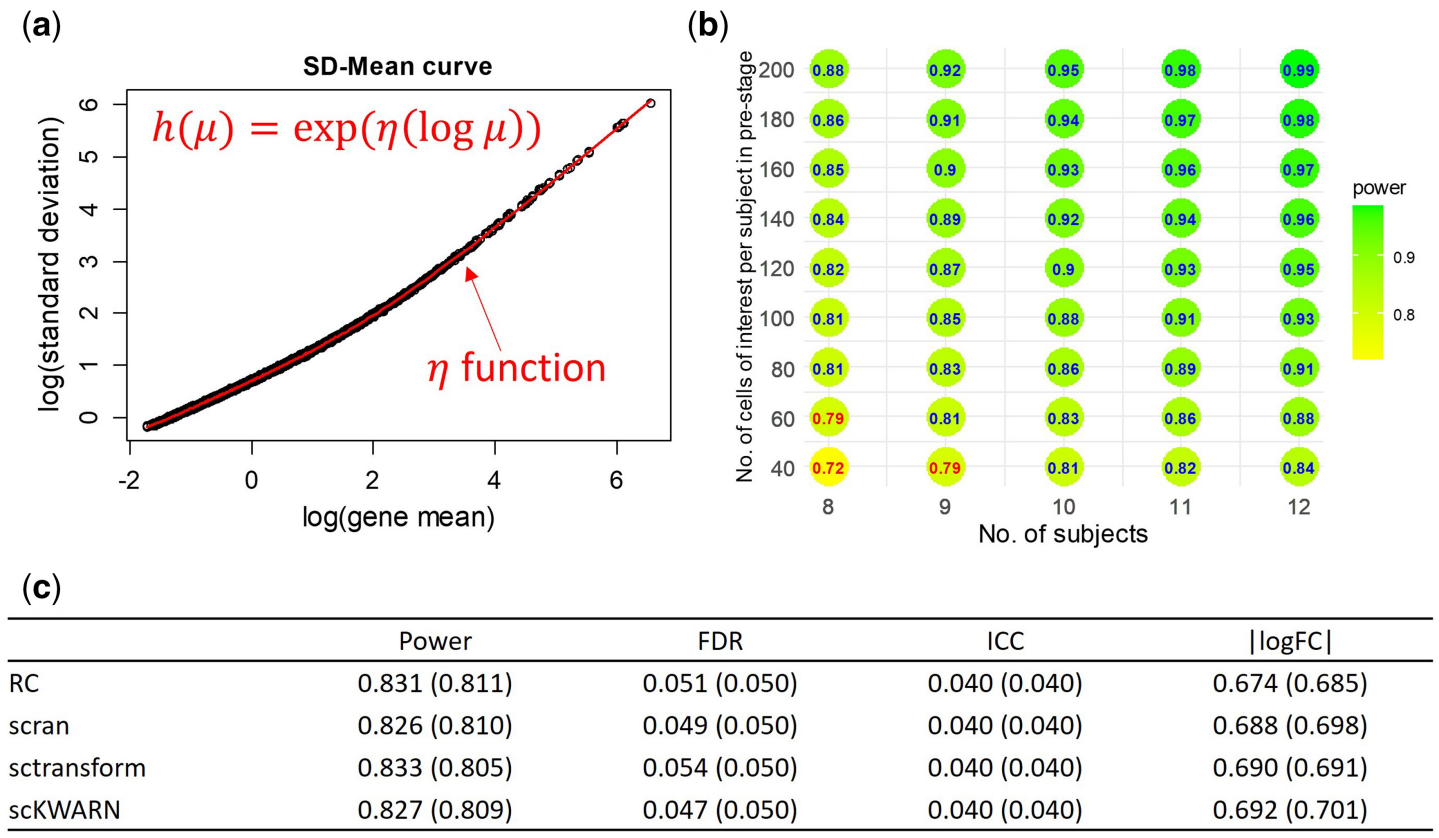
scPS estimations in a paired-group comparison. (a) Estimation for h function by fitting a composite function on means and SDs of the normalized data. (b) Required sample sizes and cells per sample to achieve a power of 0.80 under FDR = 0.05, based on the parameters estimated from the normalized data. (c) Average empirical overall power, FDR, ICC, |logFC| in 100 simulations, given 9 samples and 60 cells per group per sample. The values in the parentheses are the estimations by scPS

Next, we simulated the scenario with a paired-group experimental design, maintaining the same simulation settings. Under this design, with 9 samples and 60 cells per group per sample, scPS demonstrated a power of 0.811 under an FDR of 0.05 when RC was used (Fig. 3b). The required parameters for scPS, estimated from the RC-normalized data, included an ICC of 0.040, |logFC| of 0.685, and the h function as illustrated in Fig. 3a. In comparison, through 100 simulations, a power of 0.831 was achieved and an FDR of 0.051 was observed, closely mirroring the performance of scPS (Fig. 3c). Meanwhile, scPS estimation maintained alignment with simulation results when other three normalization methods were utilized in the paired-group design.

It’s noteworthy that the same simulation settings led to varying power outcomes when different normalization methods were applied. scPS is not tied to a specific normalization method, as it directly estimates parameters from normalized data. This flexibility allows users to choose the normalization method that best suits their data while maintaining the robustness of scPS estimation.

Furthermore, we expanded simulation to 16 scenarios, which involved two different fold changes (FC = 2 or 1.5), two correlation levels (ICC = 0.01 or 0.04), and two expression levels (normal or low) in the two experimental designs (Case 1 in Supplemental Table S3). The results demonstrated that scPS estimation was supported by simulations, with discrepancies of 3% or less observed in 12 out of 16 scenarios. In both the independent two-group and paired-group comparisons, the largest discrepancies occurred in the scenario with low expression level at FC = 1.5 and ICC = 0.04, which were 6.4% and 7.2%, respectively (Supplemental Tables S4 and S5).

Finally, we investigated the relative contribution of sample sizes and cell numbers in maintaining the power when ICC increased. We simulated six scenarios involving three correlation levels (ICC = 0.01, 0.04, 0.07) in the two experimental designs (Case 2 in Supplemental Table S3), where the ICC of 0.07 is very high based on real datasets (Supplemental Table S1). We calculated the required sample sizes and cells to achieve a power of 0.8 under FDR = 0.05. We found that having more samples is much more important than having more cells when the correlation increases. For example, in the independent two-group comparison, when the number of samples is 15, 20, 40, and 400 cells are required to achieve a power of 80% under FDR = 0.05 at the ICC = 0.01, 0.04, and 0.07, respectively. In contrast, when the number of cells is 160, 6, 11, and 16 samples are required to achieve the same power at the three ICC levels, respectively (Supplemental Fig. S2). That is, 20 times more cells are required to achieve the same power when the correlation increases from 0.01 to 0.07, while less than 3 times more samples are needed. This observation indicates that increasing the number of samples is more efficient than increasing the number of cells when the correlation is high.

### 3.2 scPS outperforms other power analysis methods

We compared scPS with three power analysis methods: scPower, powsimR, and Hierarchicell across 54 scenarios. Power analysis methods can be combined with different DE methods for downstream analysis. We evaluated scPower with edgeR (Robinson et al. 2010), powsimR with edgeR and limma (Ritchie et al. 2015), and Hierarchicell with MAST (McDavid *et al.* 2024). Performance was assessed by the biases between empirical powers (from DE methods) and expected powers (from power analysis methods).

The 54 scenarios were generated from three levels of ICC, three types of data distributions, and three combinations of sample sizes and cell numbers in two experimental designs (independent and paired) (Case 3 in Supplemental Table S3). In each scenario, each cell had 500 genes, with 3 genes being differentially expressed with FC = 1.5. The three ICC levels were set to 0, 0.01, and 0.04, reflecting no correlation, mean, and 90th quantile of ICC in the real data (Supplemental Table S1). Correlated NB and zero-inflated NB data within a sample were generated using a Gaussian copula (see details in the section “Generation of single-cell transcriptomics data with intra-sample correlations” in Supplemental materials). The three types of data distributions included one NB and two non-NB distributions: Zero-inflated NB with 0.4 extra zero proportions (LZINB) and zero-inflated NB with 0.6 extra zero proportions (HZINB). The means of the two non-NB distributions were the same as that of the NB distribution, with a mean of 1 (Supplemental Fig. S3). The three combinations of sample sizes and cell numbers had the same total number of cells (600 cells per group), including $m^{*}$=10 and $n^{*}$= 60, $m^{*}$= 20 and $n^{*}$= 30, and $m^{*}$= 30 and $n^{*}$= 20. It is important to note that scPower was excluded from the paired-group comparison since it could not be applied in that context. We included sample ID in edgeR and limma models to handle paired-group comparisons.

**Figure 4.**
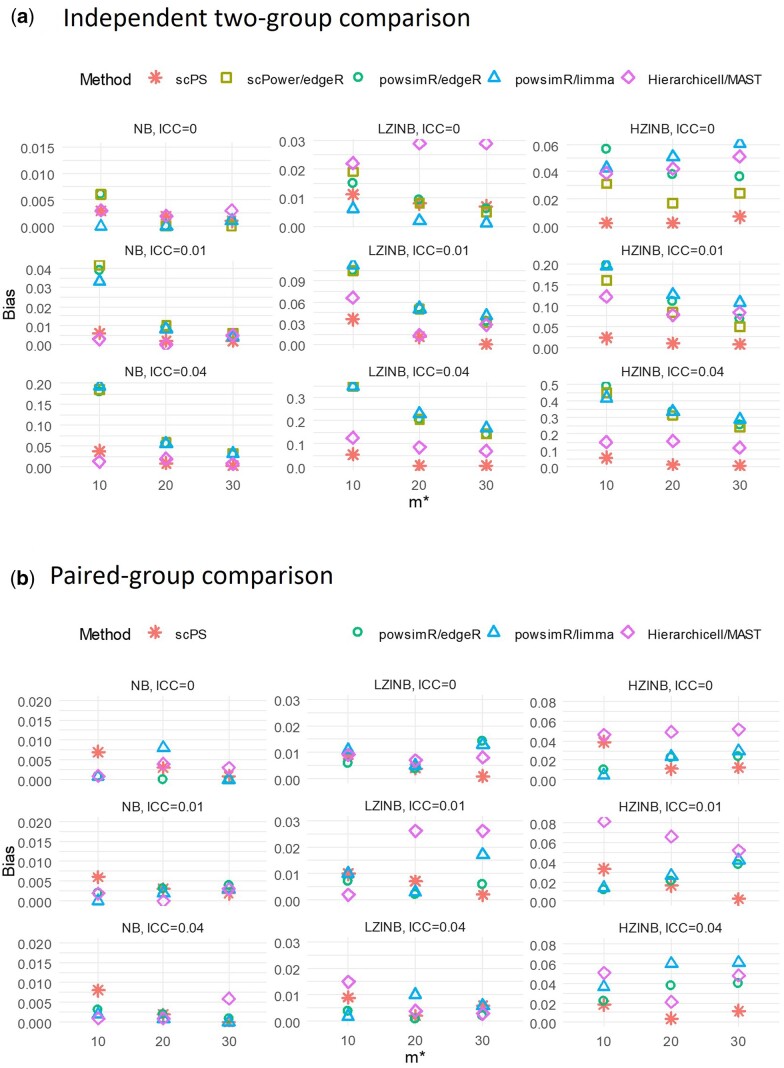
Performance comparison between scPS, powsimR, Hierachicell, and scPower. The performance was compared on the biases between expected powers (from power analysis methods) and empirical powers (from DE methods). Bias = |expected power - empirical power|. (a) Independent two-group comparison; (b) Paired-group comparison. In independent two-group comparison, $m=2m^{*}$ and $n=n^{*}$; in paired-group comparison, $m=m^{*}$ and $n={2n}^{*}$

Overall, scPS obtained the highest accuracy in power estimation. In contrast, powsimR, Hierarchicell, and scPower either overestimated or underestimated power when cell–cell correlations were present and/or data did not follow NB distributions (Fig. 4). When the data followed NB, all methods obtained low biases at ICC = 0 (biases <0.01). When ICC increased, scPS and Hierarchicell, the only two methods considering cell–cell correlations, achieved the lowest biases. In contrast, scPower and powsimR had the highest biases, especially when sample sizes were small (bias >0.03 at $m^{*}$= 10 and ICC = 0.01, bias >0.15 at $m^{*}$= 10 and ICC = 0.04). When the data did not follow NB (LZINB or HZINB), scPS had the lowest biases since it didn’t assume data should follow specific distributions. In contrast, the other three methods, which all assumed data should follow NB, obtained much higher biases (Fig. 4).

Additionally, we compared scPS and other methods in terms of computational time. As expected, the two analytic methods, scPS and scPower, are much faster than simulation-based methods (Supplemental Fig. S4).

### 3.3 scPS estimation on the COVID-19 dataset for independent two-group comparison

**Figure 5.**
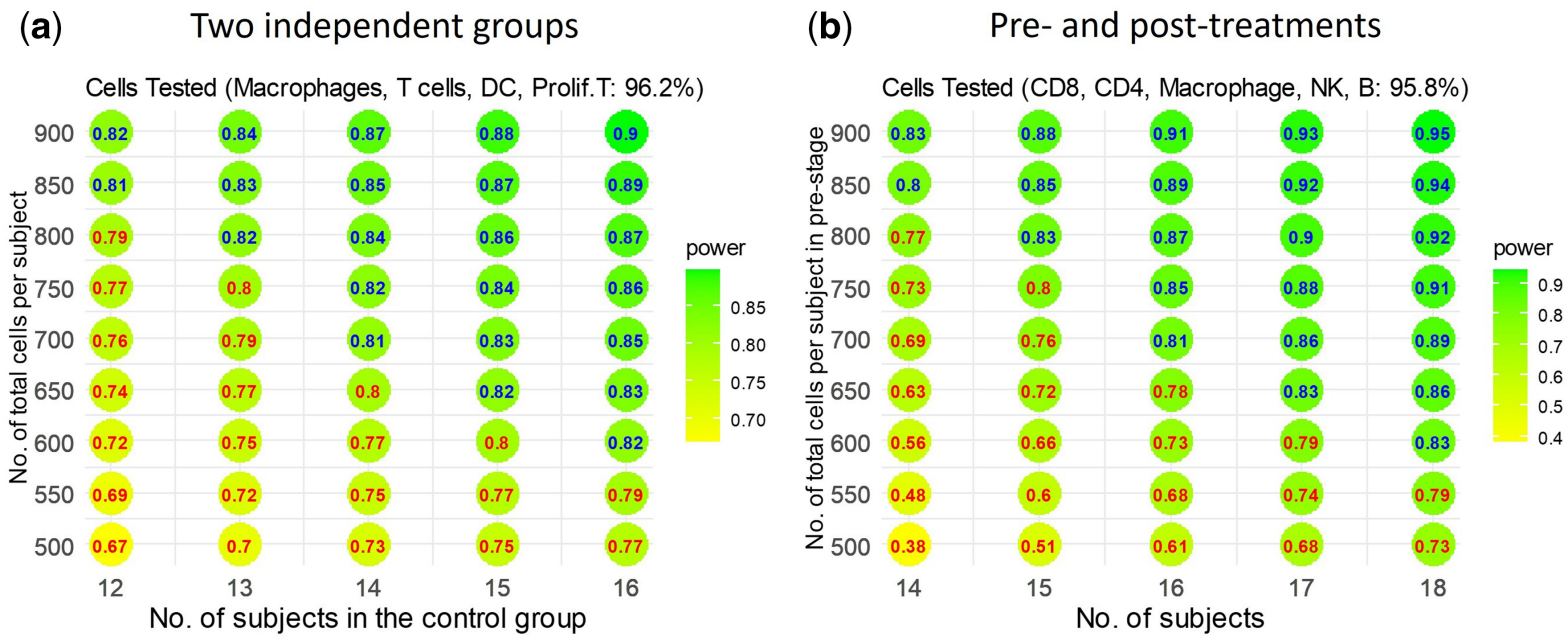
scPS estimations on the sample size and total cells per sample to achieve a power of 0.80 under FDR = 0.05. (a) To test four cell types: Macrophages, T cells, DC, and proliferating cells between two independent groups using the COVID-19 data. (b) To test five cell types: CD8, CD4, Macrophages, NK, and B cells between pre- and post-treatment groups using the checkpoint immunotherapy data

**Figure 6.**
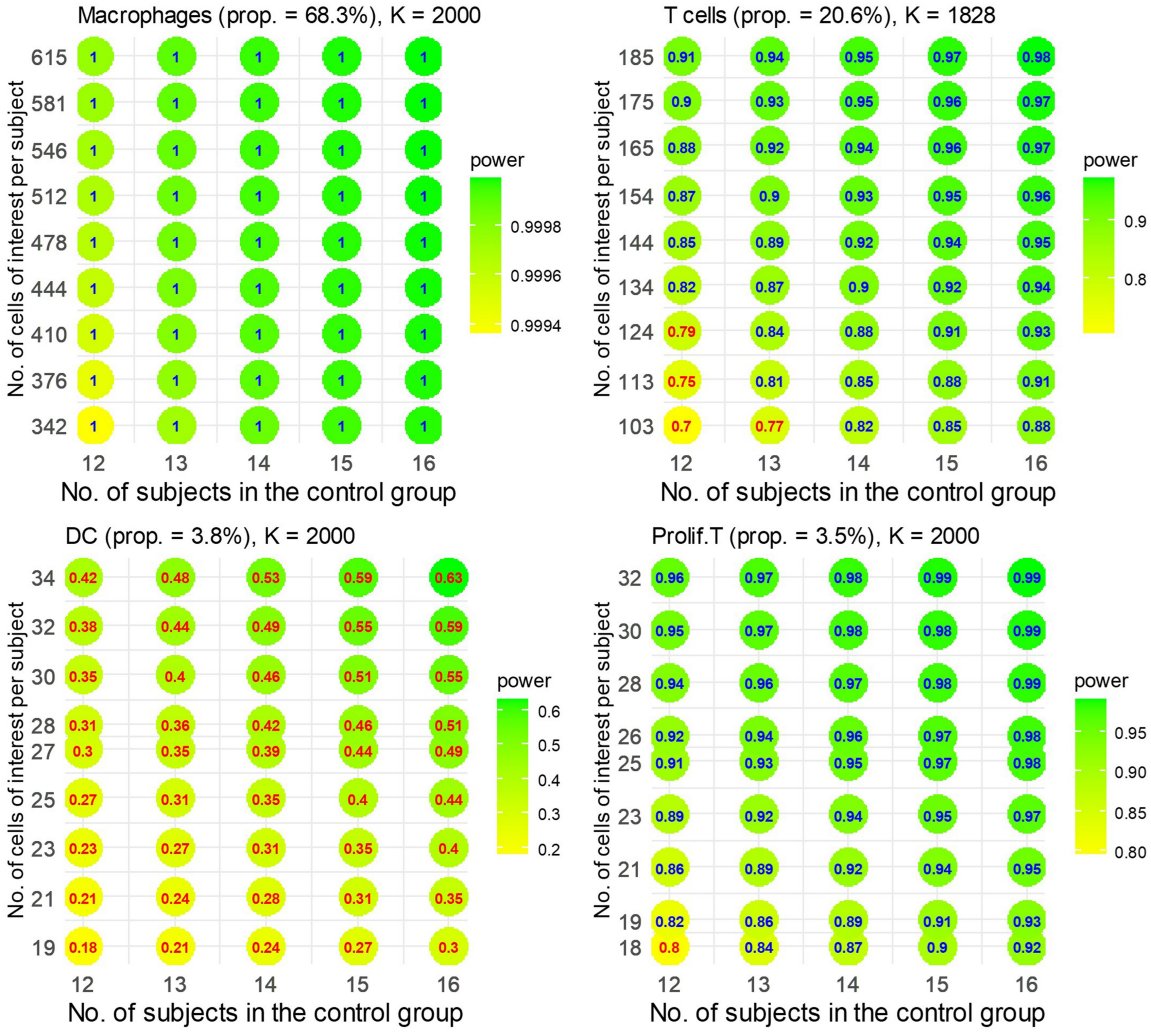
Individual power for each cell type in the independent two-group comparison. The numbers of cells are calculated by cell proportions on the total of cells in Fig. 5

We utilized scPS on the COVID-19 dataset (Grant *et al.* 2021) for the design of an independent two-group comparison, involving two patients without and five patients with COVID-19 infection. The dataset comprises 18 337 genes and 33 000 cells, spanning across seven cell types: Macrophages (68.3%), T cells (20.6%), Dendritic cells (DC) (3.8%), Proliferating T cells (3.5%), Ciliated (0.9%), Alveolar (0.5%), and Unknown (2.5%). We focused on the four cell types with larger proportions: Macrophages, T cells, DC, and Proliferating T cells. We normalized the data using the RC method. For each cell type, we identified up to 2000 candidate genes based on the following criteria: genes that were (i) expressed in more than 10% of cells in both groups and (ii) the top 2000 genes with the largest absolute values of log ratios of gene means between the two groups. This process led to 7828 candidate genes in total. The DEGs for each cell type were identified as the top 1% of genes with the smallest *P*-values among the candidate genes for that specific cell type. The *P*-values were calculated using $2\left(1-t_{m-2}\left(\left|S\right|\right)\right)$, as outlined in the Section 2.7. For power and sample size calculation, $\beta_{g0}$, $\rho_{g}$, and h were estimated through the methods detailed in the Section 2.6. Additionally, $\beta_{g1}$ was assumed to be $d\times \mathrm{sign}({\mathrm{logFC}}_{g})$, where d represented the median of the absolute values of the log ratios of mean expression among the DEGs, and ${\mathrm{logFC}}_{g}$ denoted the log mean ratio for gene g. The values of d for the four cell types were 0.877, 0.468, 0.525, and 0.942, respectively. Using these parameters and aiming for an FDR of 0.05 and an expected power of 0.80 to detect DEGs in the four cell types, Fig. 5a illustrates the required sample size (1:1 ratio between the two groups) and the total number of cells. Various combinations of sample and cell numbers are feasible. For instance, achieving a power of 0.80 under an FDR of 0.05 is possible with either 12 patients per group and 850 cells per patient or 14 patients per group and 700 cells per patient. In the former scenario, approximately 96.2% of the 850 cells are anticipated to be the cells of interest, comprising 581 Macrophages, 175 T cells, 32 DC, and 30 proliferating cells, in line with their respective cell proportions. The individual powers for the four cell types are 1.00, 0.90, 0.38, and 0.95, constituting an overall power of 0.80 (see Fig. 6). To achieve an individual power of 0.80 specifically for DC, a total of approximately 1660 cells (=63/0.038) per patient and 12 patients in each group would be necessary (Supplemental Fig. S5a).

### 3.4 scPS estimation on the checkpoint immunotherapy dataset for pre- and post-treatment comparison

**Figure 7.**
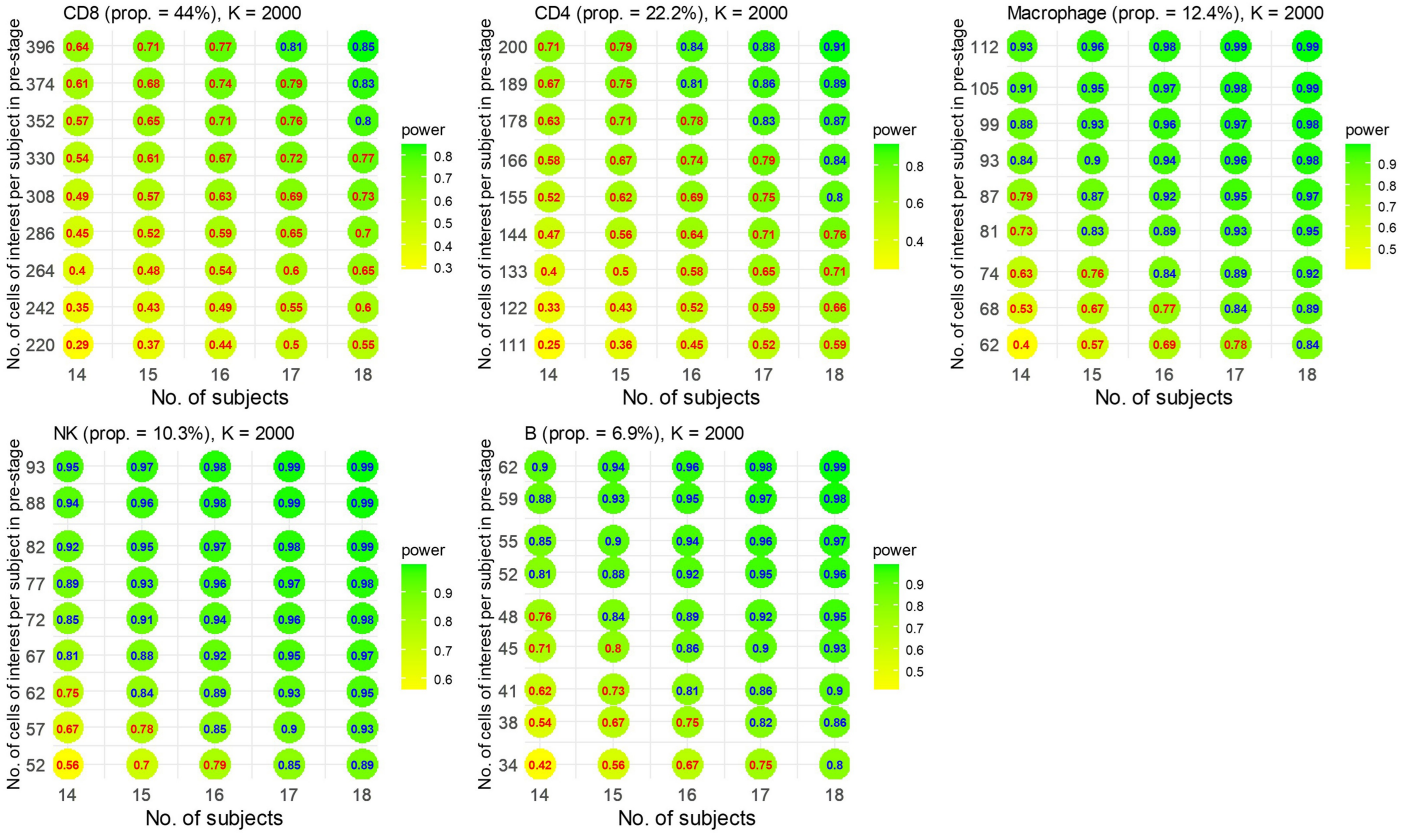
Individual power for each cell type in pre- and post-treatment comparison. The numbers of cells are calculated by cell proportions on the total of cells in Fig. 5

We utilized scPS on the checkpoint immunotherapy dataset (Sade-Feldman *et al.* 2018) for the design of a paired-group comparison, involving 11 patients measured at both pre- and post-treatment. The dataset comprises 55 736 genes and 16 291 cells, spanning across seven cell types: CD8+ cells (44.0%), CD4+ cells (22.2%), Macrophages (12.4%), Nature Killer cells (10.3%), B cells (6.9%), Plasma cells (2.4%), and DC (1.8%). We focused on the five cell types with larger proportions. We normalized the data using the RC method. We selected the candidate genes and DEGs as mentioned earlier, which led to 10 000 candidate genes and 100 DEGs in total. The values of d for the five cell types were 0.456, 0.656, 1.018, 1.078, and 1.320, respectively. Using these parameters and aiming for an FDR of 0.05 and an expected power of 0.80 to detect DEGs in the five cell types, Fig. 5b illustrates the required sample size and the total number of cells per group. Various combinations of sample and cell numbers are feasible. For example, achieving a power of 0.80 under an FDR of 0.05 is possible with 14 patients and 850 cells per group per patient. In this scenario, 95.8% of the 850 cells are expected to be the cells of interest: 374 CD8+, 189 CD4+, 105 Macrophages, 88 Nature Killer, and 59 B cells, according to their cell proportions. The individual powers for the five cell types are 0.61, 0.67, 0.91, 0.94, and 0.88, respectively (Fig. 7), constituting an overall power of 0.80. To achieve an individual power of 0.80 specifically for CD8+ cells, a total of 1200 (=528/0.44) cells per group per patient and 14 patients is necessary (Supplemental Fig. S5b).

## 4 Discussion

In this study, we proposed a novel analytical approach, scPS, employing the GEE method to compute power and determine sample sizes for differential analysis in single-cell transcriptomes. scPS presents several advantages: (i) it begins with normalized data, without being confined to a specific normalization method; (ii) makes no assumptions about data distribution; (iii) considers cell–cell correlation within samples; (iv) exhibits a considerably faster computational speed compared to simulation-based techniques; and (v) is applicable to the designs of both independent two-group and paired-group comparisons. scPS simplifies the design of single-cell transcriptomics experiments significantly.

The asymptotic covariance derived from the GEE approach helps to build a Wald-type statistic easily and calculate power. Nevertheless, it is computationally intensive to calculate the inverse of the correlation matrix in the asymptotic covariance when the number of cells per sample is large. scPS applies the Sherman–Morrison formula to simplify the calculation of the inverse matrix and speed up the computation of the asymptotic covariance.

It is difficult to make assumptions about the *P*-value distribution to control the FDR at a desired level in power and sample size estimation. In this study, we used Jung’s formula (Jung 2005) for the FDR calculation, where the formula only relies on the proportion of tests with a true null and the average power of all tests with a true difference at a significance level, without specifying distributions for *P*-values. An alternative strategy is to model a distribution of *P*-values from pilot data for FDR calculation (Ni *et al.* 2024).

scPS relies on the ICC structure to model cell–cell correlations within samples. The magnitude of the ICC significantly influences power estimation and sample size determination, with a higher ICC leading to decreased power and increased sample size requirements (Supplemental Fig. S2). scPS assumes that every sample shares the same ICC. We found this assumption reasonable since ICC distributions are similar across all leave-one-sample-out datasets (Supplemental Fig. S6).scPS learns ICC and mean-variance relationship from pilot data to give a robust estimation. When pilot data are unavailable, scPS assumes that the ICC follows a gamma distribution and the mean-variance relationship is derived from NB distributions, whose parameters are given by default values or provided by users. Specifically, scPS assumes that the ICC follows a gamma distribution (distributions from real data in Supplemental Fig. S1). The shape and scale parameters of this gamma distribution are derived from the mean and 95th percentile of ICCs, with default values set at mean = 0.01 and 95th percentile = 0.1, based on analysis across the five real datasets (Supplemental Table S1). Moreover, scPS assumes that the mean-variance relationship follows $h(\mu )=\sqrt{\mu (1+\phi \mu )}$, which is characteristic of NB distributions. Additionally, scPS assumes that μ (gene means in the control group) is generated from a gamma distribution. The parameters for shape and scale of the gamma distribution are determined from the mean and 95th percentile of gene means, with default values set at mean = 1 and 95th percentile = 2.5. The default ϕ is set to 3. These assumptions allow scPS to perform power and sample size calculations robustly even in the absence of pilot data. Meanwhile, we have created a web version of scPS for power and sample size calculation on GitHub (https://github.com/cyhsuTN/scPS), which is user-friendly for researchers without coding skills.

Currently, scPS is designed solely for two-group comparisons. Although extending scPS to accommodate multiple group comparisons and consider confounding factors is straightforward, simplifying matrix calculations becomes challenging when incorporating MD bias-corrected covariance (see the section “Extension to multiple groups” in Supplemental Materials). scPS can also be adapted to estimate power and sample sizes in spatial transcriptomics data. However, in spatial transcriptomics, cell–cell correlation may follow a spatial correlation structure rather than the ICC structure, rendering the ICC assumption inappropriate. Theoretically, replacing $C_{i}(\rho )$ in scPS with spatial correlation structures could capture actual cell–cell correlations. However, selecting an optimal spatial correlation structure and estimating its required parameters pose challenges. Additionally, computing the inverse matrix of an arbitrary spatial correlation structure may be time-consuming, especially with very large cluster sizes and no analytical formula for the inverse matrix. Therefore, our future extensions of scPS will focus significantly on extending it for multiple group comparisons and effectively integrating spatial information into power and sample size calculations.

## Supplementary Material

btae540_Supplementary_Data

## Data Availability

Data and practical guidance for experimental design using scPS are available at https://github.com/cyhsuTN/scPS.
