# Supplementary material for: A distribution-free and analytic method for power and sample size calculation in single-cell differential expression
Source: Bioinformatics. 2024 Sep 4;40(9):btae540. doi: 10.1093/bioinformatics/btae540 (PMC11407695; doi:10.1093/bioinformatics/btae540)
Supplement: btae540_Supplementary_Data [file btae540_supplementary_data.docx]

**Supplementary Materials**

**A distribution-free and analytic method for power and sample size calculation in single-cell differential expression**

Chih-Yuan Hsu, Qi Liu*, Yu Shyr*

Department of Biostatistics, Vanderbilt University Medical Center, Nashville, TN 37203, USA

Center for Quantitative Sciences, Vanderbilt University Medical Center, Nashville, TN 37203, USA

*Correspondence: qi.liu@vumc.org; yu.shyr@vumc.org

**Generation of scRNAseq data with intra-sample correlations**

Within a cluster/sample/subject, corelated NB and zero-inflated NB data are generated using a gaussian copula, expressed as follow:
(1) Generate data $\boldsymbol{Z}$ from a multivariate normal distribution with a zero mean and a covariance $\left( 1-\rho^{*} \right)\boldsymbol{I}+\rho^{*}\boldsymbol{J}$ with a correlation of $\rho^{*}$, i.e., $\boldsymbol{Z}\sim N(\boldsymbol{0}, \left( 1-\rho^{*} \right)\boldsymbol{I}+\rho^{*}\boldsymbol{J})$.
(2) Transform $\boldsymbol{Z}$ to uniform variables, i.e., $\boldsymbol{U}=\Phi(\boldsymbol{Z})$, ${\boldsymbol{U}=(U_{1}, \ldots,U}_{n})$, and $U_{j}=\Phi(Z_{j})$, where $\Phi$ is the CDF of standard normal distribution.
(3) Generate correlated (zero-inflated) negative binomial data via $\boldsymbol{Y}=F_{(ZI)NB}^{-1}\left( \boldsymbol{U} \right),$ $Y_{j}=F_{(ZI)NB}^{-1}(U_{j})$, where $F_{(ZI)NB}^{-1}$ is the inverse function of the CDF of (zero-inflated) negative binomial. Nevertheless, $\rho^{*}=cor(Z_{j},Z_{j^{'}} )\neq cor(Y_{j},Y_{j^{'}} )$. We need to adjust $\rho^{*}$ by simulation such that $cor(Y_{j},Y_{j^{'}} )=\rho$. $\rho$ here is ICC (intra-sample correlation).

**Extension to multiple groups**

The power calculation involves the calculation of $\boldsymbol{W}_{g}\boldsymbol{=}$ $\sum_{i=1}^{m} \boldsymbol{D}_{gi}^{T}\boldsymbol{V}_{gi}^{-1}\boldsymbol{D}_{gi}$ because of $Var\left( {\hat{\boldsymbol{\beta}}}_{g} \right)=\boldsymbol{W}_{g}^{-1}$. To reduce matrix calculation when cluster size ($n_{i}$) is large, we simplify $\boldsymbol{W}_{g}$ by algebra and the Sherman-Morrison formula, as follow:

$\boldsymbol{W}_{g}\boldsymbol{=}\sum_{i=1}^{m} n_{i}\left( \mu_{gi}, \mu_{gi}x_{i} \right)^{T}v_{gi}^{-1}(\mu_{gi}, \mu_{gi}x_{i})$,

where $\mu_{gi}(=\mu_{gji})=exp(\beta_{g0}+\beta_{g1}x_{i})$ and $v_{gi}^{-1}=\sigma_{gi}^{-2}\left( 1-\rho_{g} \right)^{-1}\left( 1-n_{i}\left( \frac{\rho_{g}}{1-\rho_{g}+n_{i}\rho_{g}} \right) \right)$ with $\sigma_{gi}=h(\mu_{gi})$. Thus, the expected power to detect $\beta_{g1}=0$ can be obtained easily.

Extending two groups to $k+1$ groups is straightforward in power calculation. This can be achieved by replacing $\mu_{gi}=\exp\left( \beta_{g0}+\beta_{g1}x_{i} \right)$ with $\exp\left( \beta_{g0}+\beta_{g1}x_{i1}+\ldots+\beta_{gk}x_{ik} \right)$ and rewriting $\boldsymbol{W}_{g}\boldsymbol{=}\sum_{i=1}^{m} n_{i}\left( \mu_{gi}, \mu_{gi}x_{i1},\ldots,\mu_{gi}x_{ik} \right)^{T}v_{gi}^{-1}(\mu_{gi}, \mu_{gi}x_{i1},\ldots,\mu_{gi}x_{ik})$, where $x_{il}=1$ if subject $i$ is in group $l+1$; $=0$ otherwise, $l=1,\ldots,k$. The remaining work involves assuming multiple fold changes and determining the hypotheses used for handling multiple group comparisons. In the hypothesis testing, besides calculating $\boldsymbol{W}_{g}$, it also needs the calculation of $\sum_{i=1}^{m} \boldsymbol{D}_{gi}^{T}\boldsymbol{V}_{gi}^{-1}cov\left( \boldsymbol{Y}_{gi} \right)\boldsymbol{V}_{gi}^{-1}\boldsymbol{D}_{gi}$. This can be simplified as follows:

$\sum_{i=1}^{m} \boldsymbol{D}_{gi}^{T}\boldsymbol{V}_{gi}^{-1}cov\left( \boldsymbol{Y}_{gi} \right)\boldsymbol{V}_{gi}^{-1}\boldsymbol{D}_{gi}\boldsymbol{=}\sum_{i=1}^{m} \boldsymbol{U}_{\boldsymbol{i}}\boldsymbol{U}_{\boldsymbol{i}}^{T}$,

where $\boldsymbol{U}_{\boldsymbol{i}}\boldsymbol{=}\left( \mu_{gi}, \mu_{gi}x_{i} \right)^{T}v_{gi}^{-1}\sum_{j=1}^{n_{i}} (Y_{gji}-\mu_{gi})$, and it can be extended to multiple groups by writing $\boldsymbol{U}_{\boldsymbol{i}}\boldsymbol{=}\left( \mu_{gi}, \mu_{gi}x_{i1},\ldots,\mu_{gi}x_{ik} \right)^{T}v_{gi}^{-1}\sum_{j=1}^{n_{i}} (Y_{gji}-\mu_{gi})$. However, when combining MD bias-corrected covariance to improve the false positive rate at small $m$, it is necessary to replace $\sum_{i=1}^{m} \boldsymbol{D}_{gi}^{T}\boldsymbol{V}_{gi}^{-1}\left( \boldsymbol{Y}_{gi}-{\hat{\boldsymbol{\mu}}}_{gi} \right)\left( \boldsymbol{Y}_{gi}-{\hat{\boldsymbol{\mu}}}_{gi} \right)^{T}\boldsymbol{V}_{gi}^{-1}\boldsymbol{D}_{gi}$ with $\sum_{i=1}^{m} \boldsymbol{D}_{gi}^{T}\boldsymbol{V}_{gi}^{-1}\left( \boldsymbol{I}_{i}-\boldsymbol{H}_{gii} \right)^{-1}\left( \boldsymbol{Y}_{gi}-{\hat{\boldsymbol{\mu}}}_{gi} \right)\left( \boldsymbol{Y}_{gi}-{\hat{\boldsymbol{\mu}}}_{gi} \right)^{T}\left( \boldsymbol{I}_{i}-\boldsymbol{H}_{gii} \right)^{-1}\boldsymbol{V}_{gi}^{-1}\boldsymbol{D}_{gi}$, where ${\hat{\boldsymbol{\mu}}}_{gi}$ is the estimate of $\boldsymbol{\mu}_{gi}$ and $\boldsymbol{H}_{gii}=\boldsymbol{D}_{gi}\boldsymbol{W}_{g}^{-1}\boldsymbol{D}_{gi}^{T}\boldsymbol{V}_{gi}^{-1}$. Simplifying the latter expression requires a significant amount of effort for multiple-group comparisons.

(a)


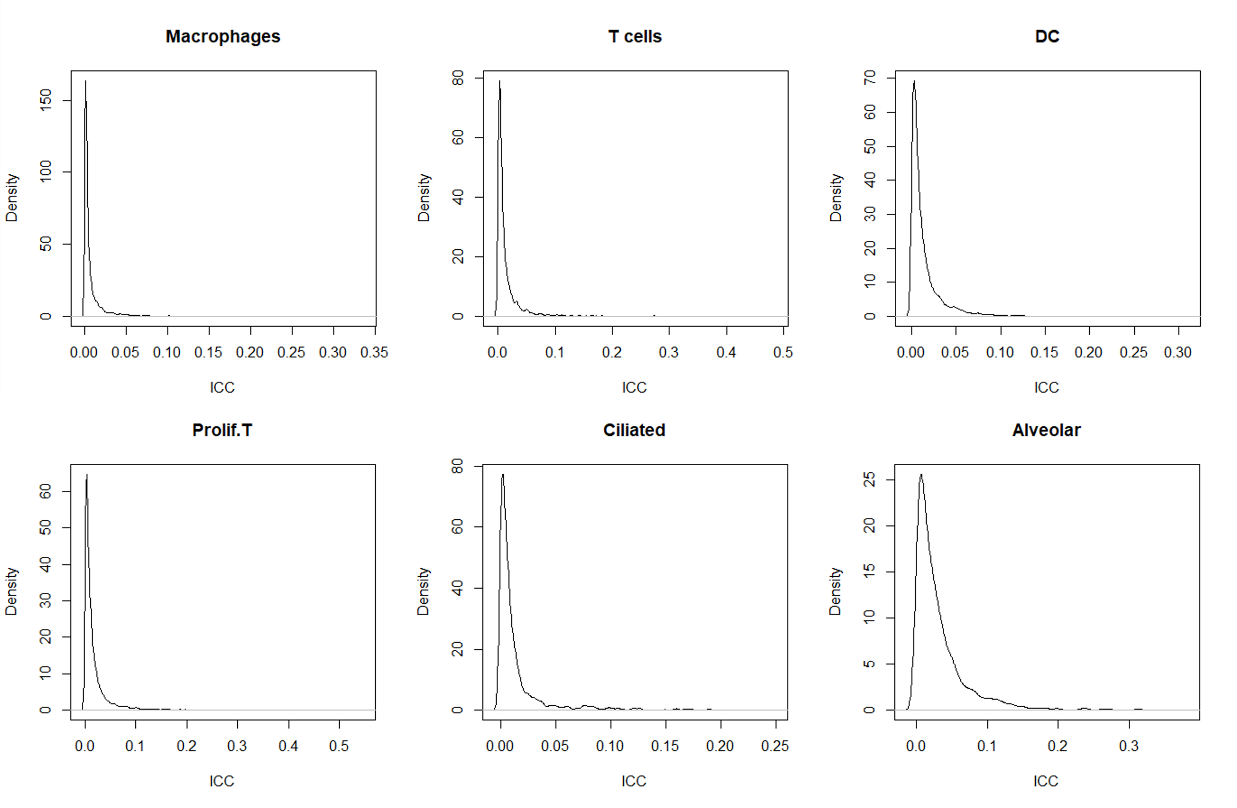


(b)


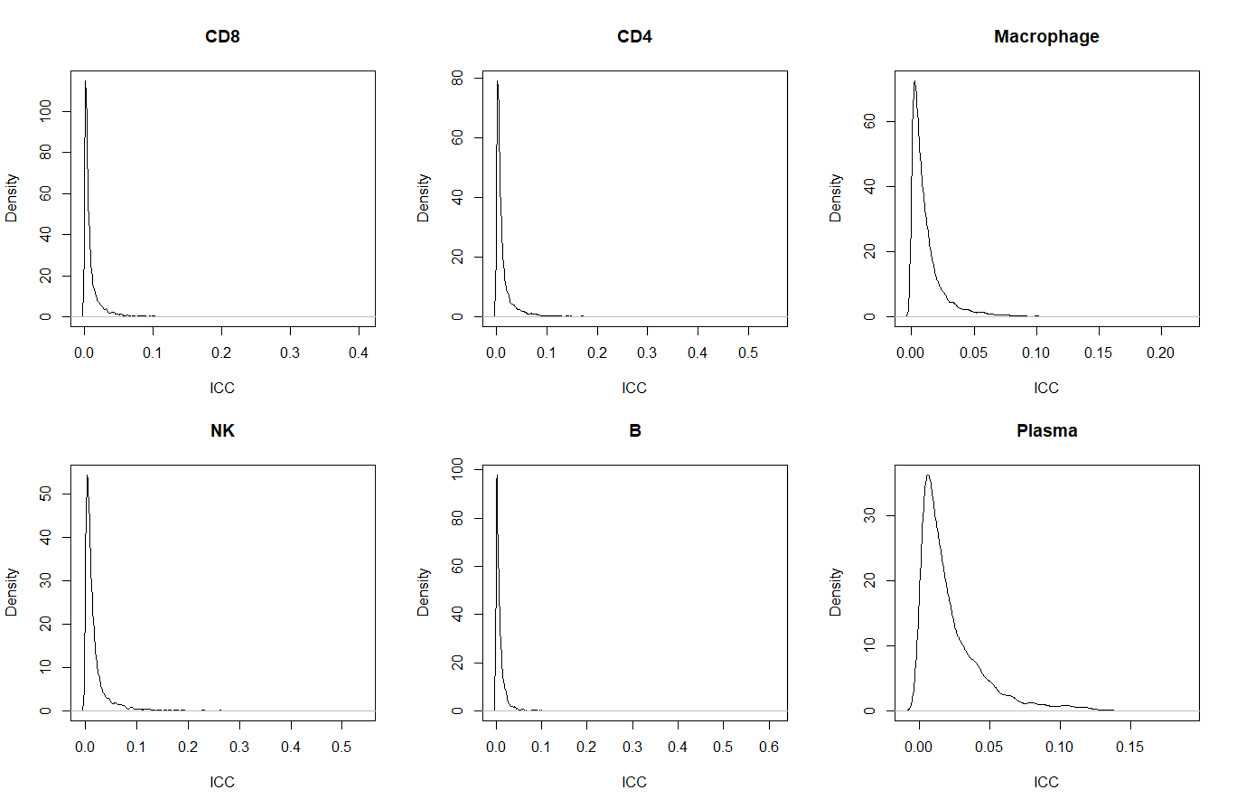


**Figure S1.** Densities of ICCs for different cell types in (a) COVID-19 data (Grant et al., 2021) and (b) GSE120575 data (Sade-Feldman et al., 2018).

**
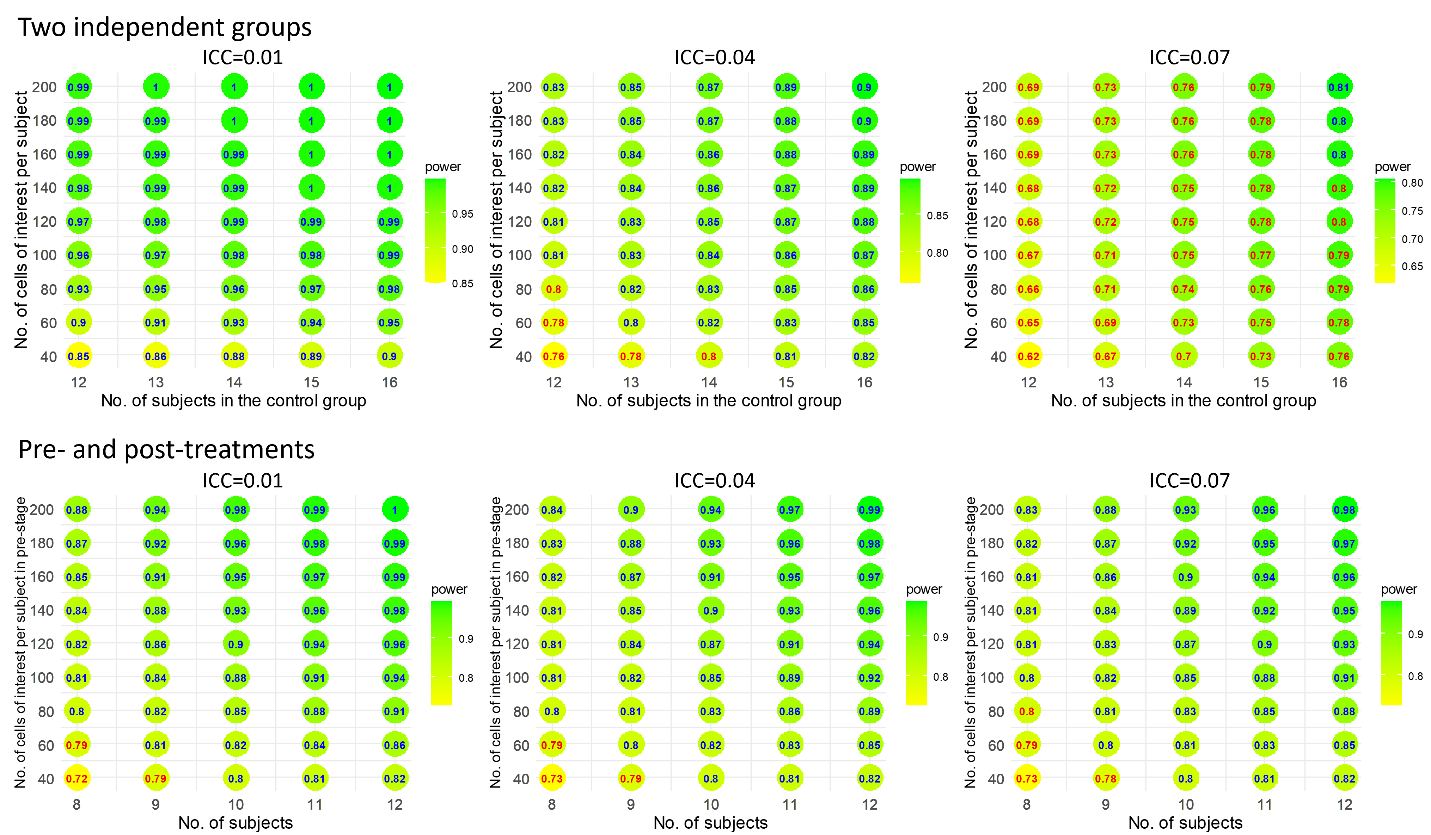
**

**Figure S2**. Required sample sizes and cells per sample to achieve a power of 0.80 (marked in blue font) under FDR = 0.05 at ICC=0.01, 0.04, and 0.07.


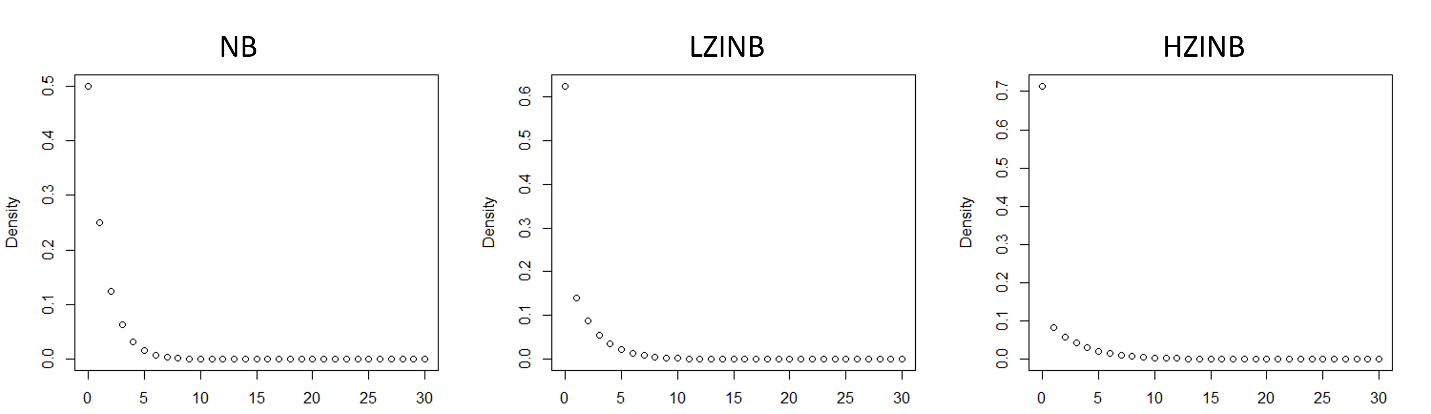


**Figure S3.** Density functions of three marginal distributions. NB: NB(mu=1, size=1); LZINB: 0.4 zero mass + 0.6 NB(mu=5/3, size=1); HZINB: 0.6 zero mass + 0.4 NB(mu=2.5, size=1). The size is the dispersion parameter.

**Figure S4.** The computation time to calculate an expected power for 500 genes with $m^{*}$=30 and $n^{*}$=20 in independent two-group comparison, when using scPS, scPower, powsimR (edgeR, 1000 simulations), powsimR (limma-trend, 1000 simulations), and Hierarchicell (MAST, 1000 simulations), via a personal computer (i7-1370P 1.90GHz, RAM 32G).

**Figure S5.** Required cells of interest per sample to achieve the 80% individual power under FDR = 0.05. (a) To test DC cells in two independent group comparison (b) To test CD8+ cells in pre- and post-treatment comparison.


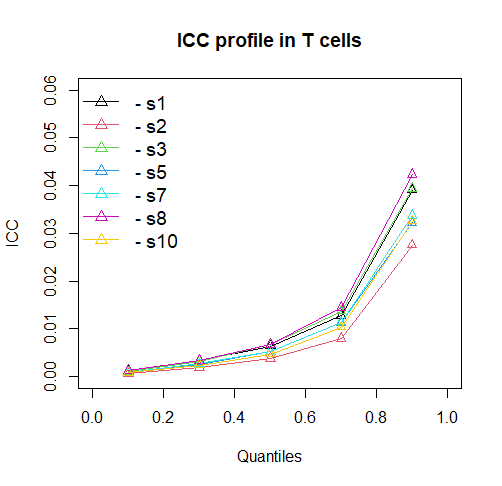


**Figure S6.** Quantile functions of ICCs in T cells in the COVID-19 dataset when one sample is left out, where -s1 is the dataset leaving the sample s1 out.

**Table S1.** Percentiles and means of the ICCs of genes in various cell types from 5 studies.

|  |  | Percentiles and mean of ICCs | | | | | | |
| --- | --- | --- | --- | --- | --- | --- | --- | --- |
| **Study (Tissue)** | **Cell type** | **10th** | **25th** | **50th** | **75th** | **90th** | **95th** | **Mean** |
| Guilliams, M. et al., 2022  (Human Liver) | T cells | 0.0052 | 0.0084 | 0.0139 | 0.0262 | 0.0530 | 0.0791 | 0.0244 |
|  | Hepatocytes | 0.0009 | 0.0035 | 0.0109 | 0.0255 | 0.0490 | 0.0703 | 0.0200 |
|  | Resident NK | 0.0051 | 0.0097 | 0.0184 | 0.0322 | 0.0577 | 0.0865 | 0.0285 |
|  | Circulating NK/NKT | 0.0080 | 0.0128 | 0.0215 | 0.0390 | 0.0755 | 0.1184 | 0.0358 |
|  | Mono+mono derived cells | 0.0028 | 0.0064 | 0.0134 | 0.0266 | 0.0484 | 0.0679 | 0.0216 |
|  | Neutrophils | 0.0001 | 0.0003 | 0.0009 | 0.0034 | 0.0120 | 0.0166 | 0.0032 |
| Stewart, B.J. et al., 2019  (Mature human kidney) | Proximal tubule | 0.0157 | 0.0213 | 0.0301 | 0.0445 | 0.0640 | 0.0843 | 0.0381 |
|  | NK cells | 0.0087 | 0.0153 | 0.0258 | 0.0412 | 0.0686 | 0.0924 | 0.0339 |
|  | Peritubular capillary endothelium | 0.0001 | 0.0004 | 0.0010 | 0.0024 | 0.0040 | 0.0060 | 0.0020 |
|  | Glomerular endothelium | 0.0001 | 0.0005 | 0.0014 | 0.0028 | 0.0053 | 0.0090 | 0.0027 |
|  | NKT cells | 0.0020 | 0.0048 | 0.0099 | 0.0203 | 0.0382 | 0.0581 | 0.0176 |
|  | CD4 T cells | 0.0056 | 0.0127 | 0.0275 | 0.0500 | 0.0932 | 0.1396 | 0.0419 |
| Grant et al., 2021  (bronchoalveolar lavage) | Macrophages | 0.0004 | 0.0011 | 0.0028 | 0.0078 | 0.0216 | 0.1091 | 0.0322 |
|  | T cells | 0.0011 | 0.0025 | 0.0058 | 0.0150 | 0.0358 | 0.0609 | 0.0159 |
|  | DC | 0.0010 | 0.0028 | 0.0071 | 0.0171 | 0.0369 | 0.0555 | 0.0512 |
|  | Proliferating T | 0.0010 | 0.0028 | 0.0076 | 0.0189 | 0.0436 | 0.0748 | 0.0192 |
|  | Ciliated | 0.0006 | 0.0020 | 0.0058 | 0.0134 | 0.0334 | 0.0667 | 0.0141 |
|  | Alveolar | 0.0028 | 0.0077 | 0.0196 | 0.0408 | 0.0778 | 0.1091 | 0.0322 |
| Sade-Feldman et al., 2018  (immune cells from melanoma biopsies) | CD8 cells | 0.0008 | 0.0018 | 0.0044 | 0.0105 | 0.0250 | 0.0417 | 0.0107 |
|  | CD4 cells | 0.0010 | 0.0026 | 0.0063 | 0.0147 | 0.0363 | 0.0583 | 0.0149 |
|  | Macrophages | 0.0012 | 0.0031 | 0.0073 | 0.0150 | 0.0286 | 0.0428 | 0.0124 |
|  | NK cells | 0.0017 | 0.0043 | 0.0094 | 0.0202 | 0.0466 | 0.0763 | 0.0207 |
|  | B cells | 0.0006 | 0.0019 | 0.0048 | 0.0106 | 0.0204 | 0.0301 | 0.0093 |
|  | Plasma cells | 0.0028 | 0.0066 | 0.0145 | 0.0298 | 0.0512 | 0.0695 | 0.0222 |
| Kanemaru, K. et al., 2023  (Human heart:Sino-atrial node region) | Fibroblast | 0.0015 | 0.0033 | 0.0077 | 0.0186 | 0.0400 | 0.0614 | 0.0162 |
|  | Atrial Cardiomyocyte | 0.0006 | 0.0016 | 0.0040 | 0.0106 | 0.0257 | 0.0429 | 0.0106 |
|  | Myeloid | 0.0050 | 0.0102 | 0.0244 | 0.0566 | 0.1051 | 0.1458 | 0.0431 |
|  | Endothelial cells | 0.0010 | 0.0026 | 0.0060 | 0.0140 | 0.0311 | 0.0502 | 0.0132 |
|  | Lymphoid | 0.0006 | 0.0014 | 0.0033 | 0.0083 | 0.0181 | 0.0382 | 0.0100 |
|  | Mural cells | 0.0014 | 0.0033 | 0.0080 | 0.0201 | 0.0491 | 0.0774 | 0.0190 |

**Table S2.** Comparison of empirical powers and FDRs between a modification with and without MD bias-corrected covariance estimator (Mancl and DeRouen, 2001). The average empirical overall powers and FDRs in 100 simulations using the same setting as described in Result section.

|  | Independent two-group comparison | | Paired-group comparison | |
| --- | --- | --- | --- | --- |
|  | Power  (wo MD vs w MD) | FDR  (wo MD vs w MD) | Power  (wo MD vs w MD) | FDR  (wo MD vs w MD) |
| RC | 0.816 vs 0.784 | 0.075 vs 0.035 | 0.831 vs 0.809 | 0.051 vs 0.020 |
| scran | 0.818 vs 0.794 | 0.069 vs 0.034 | 0.826 vs 0.807 | 0.049 vs 0.016 |
| sctransform | 0.816 vs 0.789 | 0.072 vs 0.029 | 0.833 vs 0.814 | 0.054 vs 0.026 |
| scKWARN | 0.819 vs 0.795 | 0.072 vs 0.029 | 0.827 vs 0.807 | 0.047 vs 0.018 |

**Table S3**. Scenario settings in simulation studies.

| Case (scenarios) | FC | | ICC | | | | Distribution | | | Expression | | Design | | Sample sizes |
| --- | --- | --- | --- | --- | --- | --- | --- | --- | --- | --- | --- | --- | --- | --- |
|  | 2 | 1.5 | 0 | 0.01 | 0.04 | 0.07 | NB | LZINB | HZINB | Normal | Low | Ind. | paired |  |
| Case 1  (2*2*1*2*2*1=16) | * | * |  | * | * |  | * |  |  | * | * | * | * | $m^{*}$=13 (ind.) or 9 (paired); $n^{*}$=60 |
| Case 2  (1*3*1*1*2=6) | * |  |  | * | * | * | * |  |  | * |  | * | * | NA |
| Case 3  (1*3*3*1*2*3=54) |  | * | * | * | * |  | * | * | * | * |  | * | * | $m^{*}$=10/$n^{*}$=60  $m^{*}$=20/$n^{*}$=30  $m^{*}$=30/$n^{*}$=20 |

Case 1: Comparison between analytic and empirical power of scPS

Case 2: Comparison on the effect of increasing sample sizes and cell numbers to maintain the power when ICC increases

Case 3: Comparison between scPS and other power analysis methods

Noted: The three types of data distributions included negative binomial (NB) and two non-NB distributions: zero-inflated NB with 0.4 extra zero proportions (LZINB) and zero-inflated NB with 0.6 extra zero proportions (HZINB). The means of the two non-NB distributions were the same as that of the NB distribution, with a mean of 1. In the normal expression level, mean expression values were simulated based on gene expression in proliferating T cells; in the low expression level, the mean expression values of genes were generated by dividing the normal expression level by 2. The normal expression level included 75% genes whose zero proportions were greater than 0.50, while the low expression level included 75% genes whose zero proportions were greater than 0.73.

**Table S4.** Comparison between analytic- and empirical-scPS in independent two-group comparison with 13 samples per group and 60 cells per sample. Data is normalized by the RC method. Discrepancies greater than 3% are highlighted in bold font.

| Normal expression level | | | | |
| --- | --- | --- | --- | --- |
| FC=2 | Power | FDR | ICC | \|logFC\| |
|  | .903 (.913) | .074 (.050) | 0.010 (.010) | .679 (.675) |
|  | .816 (.803) | .075 (.050) | 0.041 (.041) | .675 (.676) |
| FC=1.5 | Power | FDR | ICC | \|logFC\| |
|  | .770 (.764) | .072 (.050) | .010 (.010) | .393 (.395) |
|  | **.372 (.335)** | .077 (.050) | .041 (.041) | .397 (.393) |
| Low expression level | | | | |
| FC=2 | Power | FDR | ICC | \|logFC\| |
|  | .834 (.828) | .072 (.050) | 0.010 (.010) | .675 (.677) |
|  | .658 (.650) | .063 (.050) | 0.041 (.041) | .674 (.675) |
| FC=1.5 | Power | FDR | ICC | \|logFC\| |
|  | .615 (.604) | .061 (.050) | .010 (.010) | .397 (.405) |
|  | **.122 (.058)** | .085 (.050) | .041 (.041) | .403 (.402) |

**Table S5.** Comparison between analytic- and empirical-scPS in paired-group comparison with 9 samples and 60 cells per group per sample. Data is normalized by the RC method. Discrepancies greater than 3% are highlighted in bold font.

| Normal expression level | | | | |
| --- | --- | --- | --- | --- |
| FC=2 | Power | FDR | ICC | \|logFC\| |
|  | .832 (.812) | .039 (.050) | .010 (.010) | .675 (.684) |
|  | .831 (.811) | .051 (.050) | .040 (.040) | .674 (.685) |
| FC=1.5 | Power | FDR | ICC | \|logFC\| |
|  | .636 (.615) | .038 (.050) | .010 (.010) | .399 (.401) |
|  | .651 (.622) | .046 (.050) | .040 (.040) | .392 (.400) |
| Low expression level | | | | |
| FC=2 | Power | FDR | ICC | \|logFC\| |
|  | .775 (.772) | .042 (.050) | .011 (.011) | .676 (.679) |
|  | .780 (.765) | .069 (.050) | .040 (.040) | .681 (.677) |
| FC=1.5 | Power | FDR | ICC | \|logFC\| |
|  | **.455 (.398)** | .035 (.050) | .011 (.011) | .398 (.386) |
|  | **.484 (.412)** | .060 (.050) | .040 (.040) | .396 (.384) |

**References**

Grant RA, Morales-Nebreda L, Markov NS et al.; NU SCRIPT Study Investigators. Circuits between infected macrophages and T cells in SARS-CoV-2 pneumonia. *Nature* 2021;590:635–41.

Guilliams M, Bonnardel J, Haest B et al. Spatial proteogenomics reveals distinct and evolutionarily conserved hepatic macrophage niches*. Cell* 2022;185:379–96.e38.

Kanemaru K, Cranley J, Muraro D et al. Spatially resolved multiomics of human cardiac niches. *Nature* 2023;619:801–10.

Mancl LA, DeRouen TA. A covariance estimator for GEE with improved small-sample properties. *Biometrics* 2001;57:126–34.

Sade-Feldman M, Yizhak K, Bjorgaard SL et al. Defining T cell states associated with response to checkpoint immunotherapy in melanoma. *Cell* 2018;175:998–1013.e20.

Stewart BJ, Ferdinand JR, Young MD et al. Spatiotemporal immune zonation of the human kidney. *Science* 2019;365:1461–6.
